# Oncolytic poliovirus directs tumor antigen presentation and T cell activation *in vitro*

**DOI:** 10.1186/2051-1426-3-S2-P332

**Published:** 2015-11-04

**Authors:** Michael C Brown, Eda Holl, David Boczkowski, Ross Walton, Darell D Bigner, Matthias Gromeier, Smita K Nair

**Affiliations:** 1 Duke University, Durham, NC, USA

## Background

Our group has developed an oncolytic poliovirus, PVSRIPO, capable of lysing malignant cells while generating inflammation at the site of the tumor. PVSRIPO is a recombinant polio:rhinovirus chimera, engineered to eliminate neuropathogenicity, that has cancer tropism due to ectopic expression of the poliovirus receptor, CD155, in solid cancers. Importantly, PVSRIPO therapy is effective in the presence of neutralizing antibodies and an innate antiviral response. A first-in-human Phase I study with PVSRIPO has shown remarkable promise in patients with recurrent glioblastoma (GBM), a uniformly lethal disease. PVSRIPO tumor cell killing is associated with the induction of danger- and pathogen-associated molecular patterns (DAMPs and PAMPs) via antiviral type I interferons (IFNs) and simultaneous non-lethal infection of antigen-presenting cells (APCs) such as monocytes and dendritic cells (DCs).

## Methods

To understand key immune events associated with poliovirus infection of APCs, we examined the effects of PVSRIPO treatment on the human macrophage cell line (THP1) and primary human monocyte-derived DCs. PVSRIPO-treated DCs were evaluated for expression of maturation/activation markers and compared to untreated immature and mature DCs. To determine whether DCs exposed to PVSRIPO-induced dying tumor cells present tumor antigens to T cells, we performed an *in vitro* human assay. Human DCs generated from HLA-A2+ donor cells were incubated with PVSRIPO-induced tumor cell lysate and then used to stimulate autologous T cells *in vitro* followed by a cytotoxic T lymphocyte (CTL) assay. The following HLA-A2 human cell lines were used for this study: DM6 (MART+) melanoma cell line, MDA-MB231 (CEA+) triple-negative breast cancer cell line, SUM149 (EGFR+) inflammatory breast cancer cell line and LNCaP (PSA+).

## Results

PVSRIPO infection of THP1 macrophages and human DCs is sublethal; induces MHC class II and costimulatory molecule expression; and leads to IFN-β, IL-12, and TNF-α production. Coculturing of DCs with PVSRIPO-induced tumor lysate stimulates DC activation and IL-12 production. Human DCs loaded with PVSRIPO-induced tumor cell lysate *in vitro*, are capable of stimulating tumor antigen-specific T cells. Autologous DCs transfected with RNA that encodes for CEA, EGFR, MART or PSA were used to assess tumor antigen-specificity of T cells.

## Conclusion

Our data suggests that along with destruction of the primary tumor, oncolytic poliovirus mediates immune events. We demonstrate that human DCs co-incubated with PVSRIPO-induced tumor cell lysate stimulate tumor antigen-specific T cell responses in an *in vitro* human immunotherapy assay. In ongoing studies we are analyzing oncolytic poliovirus mediated immune events in syngeneic, immunocompetent murine models.

